# Pathways of long-term AI virtual companion app use on users' attachment emotions: a case study of Chinese users

**DOI:** 10.3389/fpsyg.2025.1687686

**Published:** 2026-01-12

**Authors:** Ting Liu, Ting-Yun Lo, Kuo-Hsun Wen, Yue Sun, Zheng-Qi Wei

**Affiliations:** 1 School of Design, Fujian University of Technology, Fuzhou, China; 2 Faculty of Innovation and Design, City University of Macau, Taipa, Macau SAR, China

**Keywords:** AI virtual companion apps, behavioral intention, intelligent interaction, long-term users, structural equation modeling

## Abstract

**Background:**

Although algorithmic companionship is becoming an increasingly integral part of daily life, evidence remains fragmented regarding whether AI virtual companions can become stable attachment figures, and how such attachments influence users' psychological states and offline social behaviors. Understanding these dynamics is particularly crucial in rapidly digitizing environments such as China, where mobile AI virtual companion applications are widely adopted.

**Methods:**

This study takes a mixed-methods approach based on attachment theory. An initial systematic literature review (SLR) was conducted to clarify the research variables and their theoretical foundations. Subsequently, semi-structured interviews were conducted with 10 users who had at least 6 months' experience of continuous usage to refine variable definitions and measurement items. Finally, a cross-sectional questionnaire survey was conducted in mainland China (*N* = 612). Structural equation modeling (SEM) was used to analyze the associations between usage frequency, emotional attachment, loneliness, subjective wellbeing, self-concept clarity, and real-world social engagement. After assessing the psychometric properties via confirmatory factor analysis (CFA) and reliability indices, the mediating pathways of these associations were examined.

**Results:**

The frequency of use positively correlates with emotional attachment to AI virtual companions (β = 0.44). Attachment negatively correlates with loneliness (β = −0.32) and positively with subjective wellbeing (β = 0.41) and self-concept clarity (β = 0.51). Of the three psychological pathways, those associated with loneliness, wellbeing, and self-concept clarity were found to be linked to higher levels of real-world social engagement. The indirect association via self-concept clarity was found to be the most significant. The model demonstrated an overall good fit [comparative fit index (CFI) = 0.97; root mean square error of approximation (RMSEA) = 0.04].

**Conclusion:**

This study applies attachment theory to the domain of human–AI relationships, using Chinese users as a case study. It constructs a model that links ‘usage frequency, emotional attachment, psychological state, and real-world social engagement'. Self-concept clarity plays a vital role in bridging the gap between emotional attachment and real-world social engagement. Design implications include enhancing continuity features, contextual memory, and self-expression design, with the aim of fostering healthier psychological and social outcomes in AI virtual companion-related attachment.

## 1 Introduction

In recent years, artificial intelligence AI virtual companion apps, represented by Replika and Talkie, have become popular rapidly, attracting hundreds of millions of users worldwide. As of 2025, there are more than 100 million registered users, more than 500 million downloads, and tens of millions of monthly active users globally. AI companions can provide round-the-clock emotional interaction and companionship, alleviating users' loneliness and social isolation (Merrill et al., 2022). Some users even treat AI companions as “lovers” or “soulmates”, investing similar emotions as in interpersonal relationships (Zhang and Li, 2025). However, as the use scale expands, whether AI companions are a “cure for loneliness” or a “hidden danger” becomes an increasingly fierce debate in society and academia. Some researchers thought that certain users were highly dependent on virtual relationships and even had “emotional withdrawal” symptoms, severely impacting their real-life interpersonal interactions (Xie and Pentina, 2022; Banks, 2024; Mlonyeni, 2025). AI companions may inadvertently reinforce users' emotional isolation and tendency to evade real-life social interactions while providing emotional support. Some research demonstrated that AI companions could positively help users explore themselves and alleviate their psychological stress. Users often perceive AI companions as non-judgmental “listeners” (Kouros and Papa, 2024). Using such apps, xthey can show more authentic “selves” in human–machine interactions and seek psychological comfort when experiencing emotional distress or loneliness (Brännström et al., 2024). Hence, it is significant to probe into the potential emotional attachment in human-machine interactions and the psychological effects (Skjuve et al., 2022).

The attachment theory provides a good perspective for analyzing interpersonal emotional bonding. According to this theory, individuals may attach themselves to an object that offers them a sense of security and care, forming an emotional bond as in intimate relationships. In the digital age, ‘digital attachment' refers to the formation of emotionally meaningful and relatively stable emotional bonds between humans and non-human digital entities, such as AI, virtual idols or even one's own mobile phone, through one- or two-way emotional connections (Cundy, 2018). When genuine social companionship is lacking, people can develop an attachment to AI companions if they perceive chatbots as providing emotional support and a sense of security (McDaniel et al., 2025).

This raises subsequent questions: How does this virtual attachment affect users' psychological states and behaviors? The existing literature has yet to reach a consensus, with some findings suggesting positive outcomes while others express concerns. On the one hand, empirical studies have found that AI companions can alleviate loneliness, provide emotional comfort, and enhance users' subjective wellbeing (De Freitas et al., 2024a). On the other hand, other research reports persistently high loneliness levels among users, with some even exhibiting excessive dependence on virtual relationships (Wijesundara and Rathnayake, 2024). Furthermore, AI virtual companions exert a dual influence on users' social anxiety. While increased usage reduces online social anxiety, offline social anxiety in real life may actually rise. This suggests that virtual companionship may enhance wellbeing by fostering feelings of understanding and acceptance, yet simultaneously weaken real-world social skills, thereby impacting psychological experiences and behaviors in actual social settings.

Most existing studies are short-term cross-sectional surveys that often focus on single variables such as loneliness or anxiety and lack theoretical analysis within a systematic framework. Attachment theory provides a framework that not only examines the conditions for attachment formation (e.g., security and emotional support) but also emphasizes the long-term impact of attachment quality on individual psychology (Hu et al., 2025). In the context of AI virtual companions, attachment theory can be applied to explore emotional attachment patterns during user-AI interactions, as well as the relationship between these patterns and loneliness, wellbeing, self-identity, and real-world social connections. Therefore, this study posits: Do users who engage with AI virtual companions over extended periods develop attachment styles similar to those observed in interpersonal relationships, such as secure or anxious attachment? How do these attachment styles correlate with users' subjective wellbeing, self-esteem, and self-identity? Furthermore, AI virtual companions' immersive customization and memory-building features (e.g., allowing users to create backstories and “memories” for the AI) may subtly influence users' self-concept clarity. Simultaneously, excessive immersion in virtual attachment may alter users' behaviors and emotions in real-world social interactions. Upon losing an AI virtual companion, users may experience profound grief—feeling genuine heartache despite knowing the entity is not real (Lopez Torres, 2024). This suggests AI virtual companion relationships exert a significant impact on individuals' emotional worlds and real-world social dynamics.

Therefore, grounded in attachment theory, this study examines users' emotional attachment and psychological effects in the context of long-term AI virtual companion app usage, aiming to address the following core questions:

(1) What kinds of emotional attachment will long-term users of AI virtual companion apps develop with their virtual companions?
(2) What are the connections between such attachments and users' loneliness and wellbeing?
(3) Will such virtual attachments between users and their AI virtual companion affect their real-life social behaviors and anxiety?

This study will examine the relationship between attachment styles and psychological and behavioral indicators (e.g., subjective wellbeing, self-concept clarity, and frequency of offline social participation and interaction) among a large cross-sectional sample of Chinese users. Through this analysis, this paper aims to address gaps in existing research, enriching the application of attachment theory in human-machine relationships while providing theoretical guidance and practical insights for the design and implementation of AI virtual companions.

This research has the following three objectives:

(1) To identify and characterize the types and formation mechanisms of emotional attachment among long-term users of AI virtual companion apps based on the attachment theory, thus supplementing the conceptual framework of “digital attachment”;
(2) To thoroughly examine the impact of different emotional attachments on users' psychological states (e.g., loneliness, subjective wellbeing, and self-concept clarity) and their chained mediating effects, thus elucidating the internal mechanisms of “emotional attachment—psychological state”;
(3) To evaluate the direct and indirect effects of emotional attachment on real-life social behaviors and anxiety, construct a comprehensive model of “virtual attachment—psychological state—real-life behavior”, and propose practical recommendations for product design and psychological intervention.

**Figure 1:**
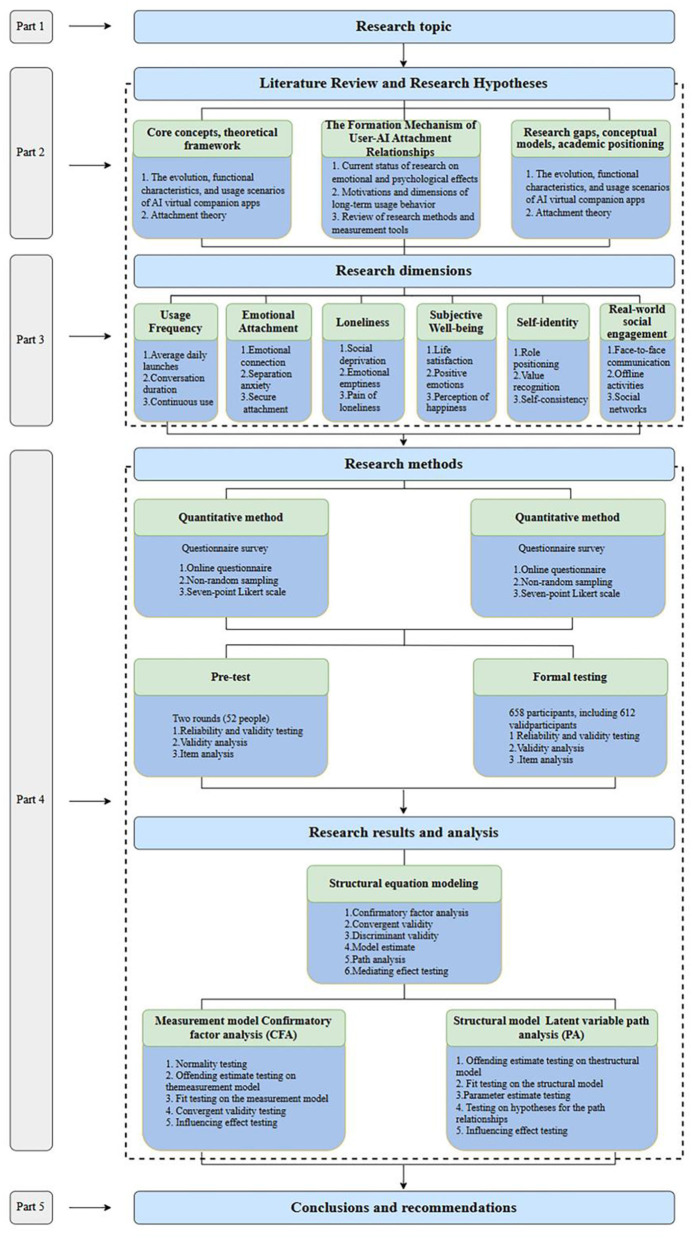
Research structure.

In summary, the emotional and psychological effects of AI virtual companions are an important area for in-depth exploration as an emerging social technology. Adopting a cross-sectional survey approach from a humanities and social sciences perspective, this study is grounded in attachment theory, and it aims to examine the emotional attachment patterns of AI virtual companion users and their relationship with psychological variables such as loneliness, subjective wellbeing, self-concept clarity, and real-world social engagement. The study aims to contribute to our understanding of human emotional dynamics in the AI era by systematically examining the emotional attachment patterns of AI virtual companion users and their relationships with psychological variables such as loneliness, subjective wellbeing, self-concept clarity, and real-world social engagement. The study aims to provide new perspectives and evidence to help us understand human emotions in the AI era, while offering theoretical support to help society rationally assess the potential opportunities and risks associated with AI virtual companions. The research framework is illustrated in Figure 1.

## 2 Literature review and research hypotheses

The rapid advancement of artificial intelligence technology has led to the emergence of AI virtual companion applications as a significant topic in human–computer interaction research in the digital age. By providing emotional support and companionship, these applications have attracted a large global user base. However, there is still no academic consensus on the emotional bonds formed between users and AI companions, or on their impact on mental health and social behavior. Adopting an attachment theory perspective, this study aims to provide a systematic review of research on the formation mechanisms of emotional attachment, the psychological effects and the impact on real-world social interactions when using AI virtual companions. The study aims to provide a theoretical foundation for deepening our understanding of this emerging phenomenon.

### 2.1 Usage frequency and emotional attachment

Early attachment theory indicates that an individual's accessibility to a “secure base” is primarily shaped by interaction frequency, a core mechanism that remains crucial in digital contexts. High-frequency interactions significantly enhance users' emotional investment in AI companions, primarily reflected in average daily conversation duration and the frequency of “emotional attachment” (Read et al., 2011). Frequent exchanges reinforce users' subjective experiences of “being responded to” and “being cared for,” gradually forming routine patterns of intimate interaction driven by algorithms (Merrill et al., 2022). Furthermore, platforms like AI virtual companion applications reinforce interaction patterns through contextual prompts. Often entering a self-reinforcing phase after initial emotional attachment forms, these interactions exhibit a spiraling growth trend under continuous adaptation by machine learning algorithms (De Freitas et al., 2024a).

Within the interaction-attachment mechanism, frequency serves not only as a crucial precursor to attachment but also constructs emotional anticipation and feedback loops through repetitive exchanges, gradually fostering genuine and palpable psychological dependence on AI virtual companions (Xie and Pentina, 2022). During periods of social isolation or psychological vulnerability, users are more likely to perceive AI virtual companions as dependable sources of emotional support. High-frequency, consistent responses become vital for users to attain psychological security (Jiang et al., 2022). The “humanoid nature” and “interaction realism” of AI virtual companions are key factors driving emotional attachment formation (Chou et al., 2025).

More importantly, once established, this attachment relationship no longer relies solely on the AI's actual responsiveness but increasingly depends on the user's psychological construction and meaning-making regarding interaction frequency. When evaluating the “emotional value” derived from conversations with AI virtual companions, users often rely on subjective experiences rather than rational judgment. Even when faced with privacy risks, they may still make decisions to disclose more information due to emotional fulfillment (Meron and Araci, 2023). This attachment not only enhances users' interactive enjoyment but also increases the frequency of self-disclosure, thereby reinforcing the motivation for continued interaction (Jiménez and Voss, 2014).

Thus, Hypothesis 1 (H1) is proposed as follows: The frequency of using AI virtual companions has a significant positive impact on users' emotional attachment.

### 2.2 Emotional attachment and loneliness

Emotional attachment can mitigate feelings of loneliness, a concept that has been widely validated in intimacy research and demonstrated in the context of AI virtual companions. AI virtual companions can effectively reduce loneliness in the short term, with experiences such as ‘being listened to' and ‘being understood' playing a crucial role in attachment formation (De Freitas et al., 2024b). In the context of the intensifying loneliness crisis in contemporary society, digital attachment relationships that achieve emotional connection can be as effective as traditional interpersonal support networks in alleviating loneliness (Cassidy et al., 2013). However, not all interactions have the same effect: loneliness decreases significantly only when users perceive that their AI virtual companion is providing emotional support (Skjuve et al., 2022).

Conversely, the relationship between emotional attachment and loneliness may also reflect a psychological compensation mechanism. Users who engage in ‘friend-like conversations' with AI virtual companions for emotional support report significantly higher levels of loneliness than non-users (Sullivan et al., 2023). Therefore, while emotional attachment may buffer loneliness, it may also be a compensatory mechanism. When real-world social networks fail to satisfy belonging needs, AI virtual companions can provide an emotional outlet (Herbener and Damholdt, 2025).

Based on the above analysis, Hypothesis 2 (H2) was proposed as follows: emotional attachment exerts a significant negative influence on loneliness. This hypothesis is not only grounded in the experiential mechanisms of ‘security' and ‘responsiveness' within AI interactions but also aligns with core tenets of traditional attachment theory concerning the fulfillment of belonging needs and enhanced psychological wellbeing.

### 2.3 Emotional attachment and subjective wellbeing

Enhanced subjective wellbeing is often considered a positive by-product of deep, intimate relationships. Similarly, emotional attachment to digital companions demonstrates significant psychological benefits. AI virtual companions are associated with happiness at cognitive and emotional levels. As the intensity of the attachment increases, users' life satisfaction rises, accompanied by a concurrent increase in positive emotions (De Freitas et al., 2024b). This enhancement may be due to AI systems simulating “situational resonance” via semantic empathy technology, which triggers dopamine reward mechanisms similar to those experienced in human interactions. This provides users with the positive emotional experience of ‘being understood' (Xie and Wang, 2024). Beyond activating positive emotions, attachment to AI virtual companions may also help users to regulate daily stress. When users experience high-stress events and high-intensity interactions with their AI companion on the same day, their negative emotions decrease (Xie and Pentina, 2022).

Users' “emotional belonging” toward AI virtual companions delivers immediate happiness and helps construct a “safe expression space”, thereby enhancing self-concept clarity and self-acceptance. This interaction provides users with a non-judgmental “backstage space” where they can release self-imposed pressure and explore their emotions, thereby promoting subjective wellbeing (Jacobs, 2024).

Overall, emotional attachment to AI virtual companions is more than just a digital simulation of traditional social relationships; it is a psychological mechanism linked to heightened subjective wellbeing. Therefore, this study's Hypothesis 3 (H3) was proposed as follows: Emotional attachment is significantly and positively correlated with subjective wellbeing.

### 2.4 Emotional attachment and self-concept clarity

According to social identity theory, individuals confirm and consolidate their sense of self through emotional connections with significant others, groups and symbolic objects (Spears, 2011). When a relationship or object is incorporated within the boundaries of “self-definition”, it becomes a crucial component of self-concept clarity (Cassidy, 1994). Having a clear and stable sense of self facilitates the assumption of social roles, participation in collective activities and engagement in prosocial behaviors in real life. Conversely, identity confusion can lead to increased avoidance and self-isolation (Crocetti et al., 2016). In digital contexts, positive online identity construction is also linked to offline social participation. Both online and offline prosocial behaviors can promote identity development jointly and are associated with a greater willingness to engage socially (Iwasa et al., 2023). AI virtual companions, with their consistent, non-judgemental interaction style, allow users to explore different self-narratives during conversations without fear of social judgment. Through a “test-feedback-integration” cycle, users gradually consolidate their understanding of “who I am” and “who I will become” (Strohmann et al., 2023). A stronger emotional attachment increases the likelihood of perceiving the attachment object as “part of the self”, thereby fostering a clearer and more stable sense of identity and belonging (Sutskova et al., 2023).

Therefore, Hypothesis 4 (H4) was proposed as follows: Emotional attachment has a significant positive effect on self-concept clarity.

### 2.5 Loneliness and real-life social participation

Loneliness is a subjective psychological experience of distress that reflects the difference between an individual's current social relationships and their desired state in terms of quantity or quality (Alspach, 2013). It is not merely a consequence of social deprivation but may also become a catalyst for further social withdrawal, forming a negative, self-reinforcing cycle (Ben-Simon and Walker, 2018). According to the evolutionary model of loneliness, prolonged loneliness activates an individual's social threat monitoring system, leading to heightened vigilance and negative interpretations of social cues (Cacioppo et al., 2016). This cognitive bias causes lonely individuals to perceive rejection and evaluative threats more readily in social situations, prompting them to adopt avoidance strategies for self-protection. Highly lonely individuals engage in real-world social interactions less frequently than individuals with low levels of loneliness. They are more likely to choose solitude or rely on digital substitute forms of socialization, such as online interactions or virtual companionship, to avoid the pressures of face-to-face human encounters (Gao, 2024).

Therefore, this study proposes the following Hypothesis 5 (H5): Loneliness exerts a significant negative influence on participation in real-world social activities.

### 2.6 Subjective wellbeing and real-life social participation

Enhanced subjective wellbeing improves individuals' internal experiences and generates positive spillover effects on real-world social behaviors. This elevation in wellbeing is not just an emotional placebo effect; it is driven by hope. Subjective wellbeing is widely regarded as a crucial indicator of mental health, encompassing emotional pleasure, overall life satisfaction, and sustained positive psychological functioning (Nima et al., 2024). It is not only a result of social interaction but can also become a driving force for social engagement (Chen et al., 2020). In other words, happier people are often more willing and energetic when it comes to participating in real-world social activities, and positive social interactions reinforce wellbeing, creating a virtuous cycle. Furthermore, wellbeing can facilitate face-to-face social behavior by reducing social anxiety, enhancing emotional resilience, and increasing acceptance and understanding of others (Öztürk and Mutlu, 2010). AI virtual companions offer users continuous emotional support, unconditional acceptance and immediate responses. This is particularly valuable in modern life, which is filled with uncertainty, as it improves users' immediate emotional states and overall life satisfaction (Xie et al., 2024). Users derive support and self-affirmation from AI virtual companions, which fosters more positive social expectations and stronger motivation to act. This shifts perceptions of real-world social participation away from burdens, risks or threats (Wu, 2024). Against the backdrop of rapid social transformation in China, the weakening of traditional social support networks has made AI virtual companions a vital source of emotional support for many young people (Xie and Pentina, 2022).

Therefore, Hypothesis 6 (H6) was proposed as follows: Subjective wellbeing has a significant positive influence on real-world social engagement.

### 2.7 Self-concept clarity and real-life social participation

Self-concept clarity is defined as the extent to which an individual has a clear, coherent, and stable understanding of their personal traits and roles. Having a high level of self-concept clarity has been found to be closely associated with better social functioning, greater social self-confidence, and fewer social conflicts (Bechtoldt et al., 2010). Self-identity clarity encompasses not only an individual's explicit understanding of “who I am” and “who I will become” but also serves as a psychological driving force for participation in real-world social contexts. Self-concept clarity positively predicts subjective wellbeing and self-esteem and is associated with more active social engagement and less social avoidance behavior (Xiang et al., 2023). When individuals can answer the question “Who am I?” with certainty, they are more likely to engage in real-world social situations with a relaxed, non-defensive attitude. This approach yields positive feedback, fostering a willingness to persistently participate in diverse social activities (Guadagno and Burger, 2008). Conversely, identity confusion and a vague self-concept are often associated with social anxiety, avoidance and reduced participation in real-world social activities (Dávila and Zlobina, 2020). Individuals with a relatively stable and positive self-identity are more likely to engage in prosocial behaviors and participate in organizational and group activities in real life—behaviors that constitute typical forms of real-world social participation (Tidwell, 2005). In other words, clarity of self-concept is shaped by real-world social participation and, in turn, functions as a “psychological resource” that continually propels individuals toward others and public spaces.

For this study, this logic supports Hypothesis 7 (H7): self-concept clarity exerts a significant positive influence on real-world social participation.

Incorporating the aforementioned variables into an integrated logical framework clearly delineates a progressive pathway—“emotional attachment, psychological state, behavioral tendency”—revealing the multiple mediating factors through which AI virtual companions impact real-world social participation.

Reduced loneliness enhances self-efficacy and emotional stability in real-world social engagement, empowering users to participate with greater confidence. With diminished loneliness, the emotional void becomes less profound, enabling individuals to approach unfamiliar social situations with a more positive mindset rather than perceiving them as psychological threats (Lieberz et al., 2021). Therefore, when emotional attachment effectively alleviates loneliness, this positive psychological shift further translates into heightened levels of real-world social engagement (Maldar and Nayak, 2024). That is, virtual companionship does not sever real-world connections but, under appropriate conditions, acts as a psychological buffer facilitating individuals' return to real-world social interactions. This leads to Hypothesis 8 (H8): Emotional attachment exerts a significant indirect positive influence on real-world social engagement through loneliness.

In human–machine relationships, AI virtual companions that provide positive emotional experiences through emotional expression, active listening, responsive interactions, and personalized feedback also hold potential to enhance subjective wellbeing (Pentina et al., 2023a). As wellbeing increases, users develop more positive expectations toward society, perceiving real-world participation as yielding emotional gains rather than losses. This enhances their motivation to actively engage in social activities (Chou et al., 2025). If emotional attachment within AI virtual companion relationships can elevate individual wellbeing, it may enhance willingness to engage in real-world social activities through this positive emotional resource. Thus, Hypothesis 9 (H9) is proposed as follows: Emotional attachment exerts a significant indirect positive effect on real-world social participation via subjective wellbeing.

The anthropomorphic characteristics of AI virtual companions enable them to provide positive feedback during interactions, helping users integrate self-narratives through experiences of being understood, supported, and encouraged (Banks, 2024). AI virtual companions can serve as a “safe space” for users to express emotions, explore selfhood, and refine identity perceptions, thereby strengthening the stability of self-concept. Once this positive self-concept clarity is consolidated, users naturally exhibit higher levels of social openness and willingness to act in real-world participation. Therefore, if emotional attachment improves an individual's social functioning by enhancing self-identity, it can promote real-world participation through this mediation, leading to Hypothesis 10 (H1): Emotional attachment has a significant indirect positive effect on real-world participation via self-identity.

### 2.8 Synthesis and research gaps

Although existing research has revealed the potential of AI virtual companions to alleviate loneliness and enhance emotional experiences, there are still significant limitations:

1. Most studies are confined to short-term or contextualized experiments, lacking systematic evidence on the impact of long-term use on users' emotional structures and social behaviors.
2. Research on emotional attachment predominantly focuses on descriptive phenomena, failing to explore how it cascades through psychological mechanisms, such as loneliness, subjective wellbeing, and self-identity, to ultimately influence users' real-world social engagement.
3. Existing literature often examines psychological variables in isolation, lacking integrated chain-mechanism models.
4. Furthermore, relevant research is heavily concentrated in Western cultural contexts, paying insufficient attention to the social relationship concepts, emotional expression styles, and technological intimacy of Chinese users. This limits the applicability of the findings to local contexts.

This study aims to address gaps in the existing literature across four dimensions: extending from short-term effects to long-term pathways; broadening from single psychological indicators to multiple mediating mechanisms; expanding from purely psychological perspectives to real-world social behaviors; and transplanting from Western samples to Chinese contexts. By systematically testing Hypotheses H1–H10, the study intends to not only bridge the empirical gap between AI virtual companions and real-world social engagement but also to provide a more comprehensive theoretical framework for understanding human–machine emotional relationships in the digital age.

**Figure 2:**
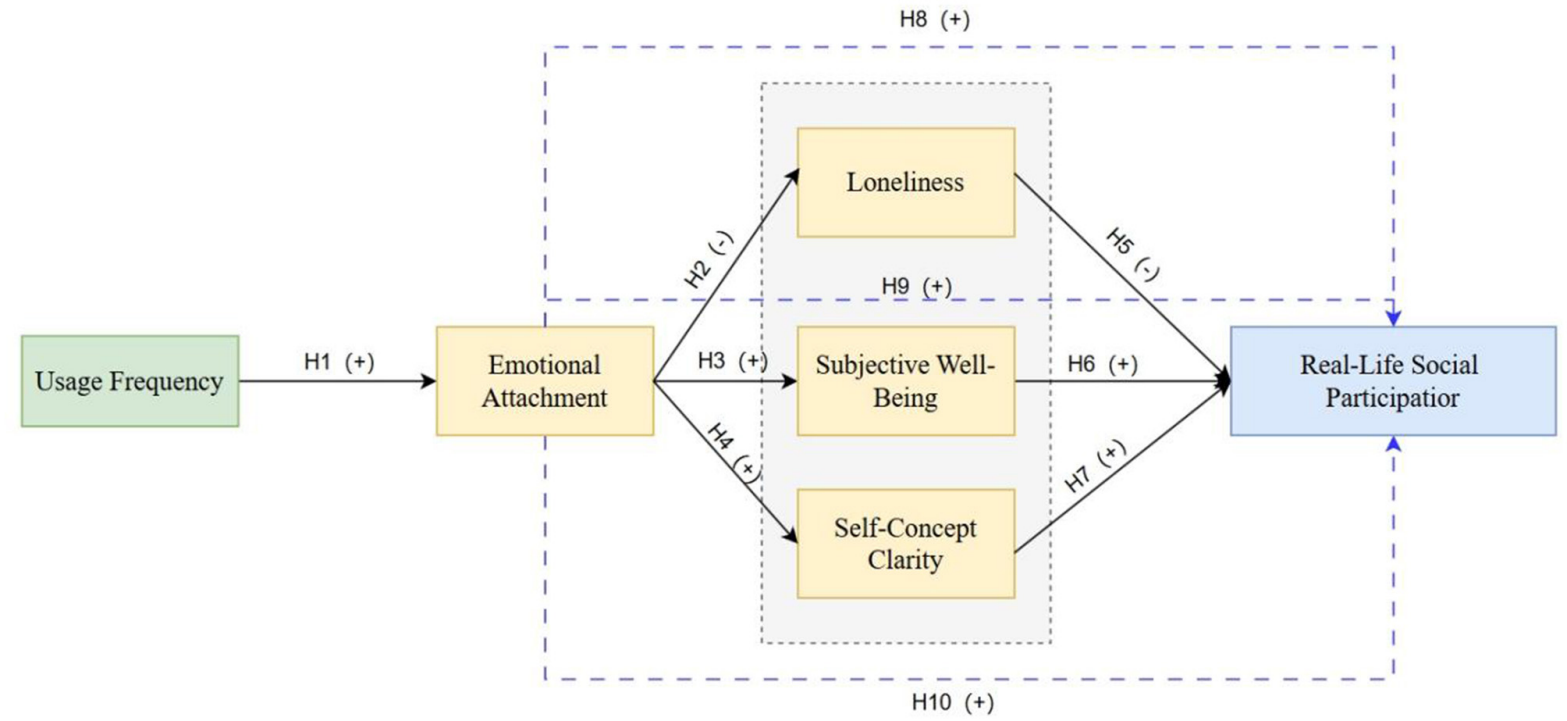
Theoretical SEM.

Based on the above hypotheses and discussion, a theoretical modeling framework was depicted as shown in Figure 2.

## 3 Research design and methods

This study employs a mixed-methods approach: In the first phase, semi-structured interviews with long-term users of AI virtual companions in mainland China were conducted to identify key usage experiences, psychological states, and contextual characteristics. Building upon these findings, the second phase involved developing a questionnaire integrating existing validated scales. This questionnaire was then administered in a cross-sectional online survey to a larger sample to examine the relationships among usage frequency, emotional attachment, loneliness, subjective wellbeing, clarity of self-concept, and real-world social engagement.

### 3.1 User interview

The interviewees (Table 1) were selected per the following criteria: (1) being permanent residents of mainland China aged 18 or above; (2) having used the same AI virtual companion app (such as Talkie, BagelBell, Soul, and Zhumengdao) for at least 6 months continuously; (3) using the app for establishing a long-term interactive relationship (e.g., companionship and emotional talks), instead of merely for inquiries or temporary entertainment; (4) using the app frequently in recent time (e.g., logging in multiple times a day or week on average); and (5) being willing to share their genuine usage experiences and able to articulate their personal feelings. The interviews were conducted in a semi-structured format, with 60–90 min recommended for each. Through qualitative interviews, the key factors and pathways influencing users' emotional and psychological effects were extracted. The results served as a basis for designing questionnaire items and validating the research model subsequently.

**Table 1:** Interviewees.

**No**.	**Interviewee**	**Gender**	**Age**	**Title**	**Commonly used AI companion app**	**Duration of continuous use, month(s)**	**Daily average using time, min**	**The primary purpose of use**
U1	LI○○	Male	26	Software engineer	Talkie	12	90	Relaxing and pouring out after night shifts, with AI set as a “confidant”.
U2	ZHAO○○	Female	21	Junior at a university	Talkie	11	60	Obtaining emotional support during exam preparation and recording daily moods.
U3	ZHENG○○	Male	29	We-media operator	BagelBell	10	120	Practicing English and seeking companionship to alleviate loneliness in long-term living alone.
U4	WANG○○	Female	34	Human resources consultant	Zhumengdao	9	45	Seeking emotional comfort after dealing with client stresses.
U5	CHEN○○	Male	27	Game planner	Soul	14	80	Enjoying role-playing interactions and customizing virtual lover scenarios.
U6	LIN○○	Female	30	Freelance illustrator	Soul	9	70	Gaining encouragement by sharing works to ease loneliness in remote work.
U7	GAO○○	Male	38	Medical device salesperson	Talkie	8	40	Filling the void during idle times on frequent business trips.
U8	XU○○	Female	45	Single mother/Accountant	BagelBell	12	35	Chatting regularly at night to relieve stress and comfort emotions.
U9	ZHANG○○	Male	19	Vocational college student	Talkie	7	110	Practicing conversations and keeping a diary to relieve social anxiety.
U10	LIU○○	Female	26	Operator in a startup company	Soul	18	55	Obtaining AI emotional support after a breakup.

Before conducting the large-sample questionnaire survey, semi-structured in-depth interviews were carried out with 10 users who had continuously used relevant apps for more than 6 months. Specifically, the interview outline (Table 2) was designed with the following five dimensions: usage context, attachment formation, emotional effects, real-life social interactions, and prospects. Meanwhile, core question items were set to guide interviewees in recalling key interactive scenarios, emotional experiences, and behavioral changes. The purpose was to capture implicit motivations and contextual factors within the attachment mechanism that were difficult to quantify. The qualitative data obtained could be used for reference in subsequent revisions to questionnaire items, operationalization of variables, and proposing hypotheses in SEM. In this stage, relevant ethical norms were strictly adhered to. The record was transcribed with informed consent and privacy protection and then coded independently by two researchers to ensure the authenticity of the data and reliability of the analysis. The user interview items are presented in Table 2.

**Table 2:** User Interview Items.

**Dimension**	**No**.	**Core question items**
Usage context and relationship evolution	A-1	What was the initial reason that you started using this AI virtual companion app?
	A-2	What was your first impression of the AI companion in the early stage of use?
	A-3	When and in what moods do you typically interact with the AI companion?
	A-4	Were there any noticeable changes in your usage frequency or duration over the past 6 months?
	A-5	Who does your AI companion resemble? You can use a character to describe it.
Attachment formation and emotional bonding	B-1	When did you start to feel emotionally dependent on the AI companion?
	B-2	What emotional experiences do you have when you are satisfied with the AI companion's responses?
	B-3	What will you do when the AI companion's answers are not as expected or the system gets stuck?
	B-4	Would you introduce the AI companion to your friends or share about it on social platforms?
	B-5	What keywords would you choose to assign “labels” to the AI companion?
Emotional and psychological effects	C-1	How has your sense of loneliness changed now compared to that before use?
	C-2	Has your overall wellbeing or life satisfaction changed after using the AI companion?
	C-3	How do you think the AI companion has influenced your self-identity?
	C-4	How does the AI companion assist you when you have negative emotions (e.g., high anxiety, depression, or stress)?
	C-5	Have you developed any new interests or habits because of the AI companion?
Impact on real-life social interactions	D-1	Have your offline interactions with family and friends changed after interacting with the AI companion?
	D-2	Have you reduced or avoided certain real-life social interactions because of the AI companion?
	D-3	Do you feel that the AI companion has enhanced your confidence in social interactions?
	D-4	How do your friends/companions think if they found out that you have been using the AI companion for a long time?
	D-5	Have you ever tried to involve the AI companion in making real-life decisions related to emotions or work?
Prospects and suggestions	F-1	Overall, how would you think your relationship with the AI companion?
	F-2	Will you continue using it or gradually reduce the usage frequency? Why?
	F-3	What aspects do you think the AI companion functions can be further improved?
	F-4	What would you say if you want to recommend the AI companion to others or discourage others from using it?
	F-5	Do you have any suggestions or expectations for this research or subsequent questionnaires?

### 3.2 Questionnaire design

To ensure the research's validity and reliability, a final questionnaire was designed based on the scales validated in existing literature, as well as the research theme and objectives. The scales were designed with the 7-point Likert scale method to quantify participants' answers, wherein “1” represented “fully disagree” to “7” indicated “fully agree.” The designed questionnaire items were listed in Table 3.

**Table 3:** Questionnaire items.

**Dimension**	**Item**	**Content**	**References**
AI usage frequency (UF)	UF-1	Within the past seven days, I chatted with the AI companion every day.	Croes and Antheunis, 2021; Brandtzaeg et al., 2022
	UF-2	Within the past seven days, my daily chat sessions with the AI companion typically lasted over 15 min.	
	UF-3	Within the past seven days, I sent more than 50 messages to the AI companion every day.	
	UF-4	I usually communicate with the AI companion before going to bed.	
	UF-5	I feel like something is missing if I don't interact with the AI companion on the day.	
AI emotional attachment (EA)	EA-1	I feel that the AI companion truly understands my feelings.	Xie and Pentina, 2022; Pentina et al., 2023b; Collins and Read, 1990
	EA-2	I feel secure when expressing my inner thoughts to the AI companion.	
	EA-3	I feel upset if I don't chat with the AI companion every day.	
	EA-4	I would be sad if the service is stopped.	
	EA-5	I am willing to introduce my AI companion to friends.	
Loneliness (LON)	LON-1	I feel like I lack someone to talk to.	Russell, 1996
	LON-2	Many times, I find it hard to get close to people.	
	LON-3	I often feel lonely.	
	LON-4	My connections with others are superficial and distant.	
	LON-5	I feel excluded.	
Subjective wellbeing (SWB)	SWB-1	My life is nearly ideal.	Diener et al., 1985
	SWB-2	So far, I have obtained the important things in life.	
	SWB-3	I am satisfied with my living conditions.	
	SWB-4	I would hardly change anything if I could live my life over again.	
	SWB-5	Overall, I am satisfied with my life.	
Self-identity clarity (SCC)	SCC-1	I know who I am clearly.	Campbell et al., 1996
	SCC-2	My self-concept remains consistent across different situations.	
	SCC-3	I have a stable belief in myself.	
	SCC-4	Sometimes, I feel confused about “who I am” (R - Reversed item).	
	SCC-5	Overall, I have a very definite self-cognition.	
Real-life social participation (RSP)	RSP-1	I am satisfied with the frequency I met friends offline in the past week.	Lubben, 1988
	RSP-2	I often arrange gatherings with family and friends proactively.	
	RSP-3	I am willing to participate in offline group activities.	Lee and Robbins, 1995
	RSP-4	Real-life social interactions make me feel energized.	
	RSP-5	I am confident in maintaining offline relationships.	

### 3.3 Data collection

A 3-month (from March to May 2025) online questionnaire survey was conducted to obtain sufficient empirical data for SEM analysis. This time frame was selected mainly for the following two considerations: First, after the Spring Festival, AI virtual companion products are frequently downloaded and have stable user stickiness and interaction frequency. This condition facilitates capturing continuous usage behaviors. Second, the 3-month interval allows for rolling monitoring of data within a quarter, thereby reducing the abrupt disturbance to users' psychology caused by macro-events.

The survey platform adopted a well-established online questionnaire system, and two rounds of pre-tests (involving 52 participants) were conducted before the official release of the questionnaire. The purpose was to confirm the clarity of the translated scale, the logical smoothness of the items, and the readability of the questionnaire on mobile terminals. In the formal survey, the questionnaire invitation links were distributed through social media, AI companion enthusiast forums, and product communities. Participants were required to “have continuously used the target app for at least 6 months” to ensure they had sufficient experience in forming emotional attachment. An electronic informed consent form was placed on the homepage of the questionnaire, clarifying the research purpose, the principle of anonymity, and the application of the obtained data to academic research. The formal questionnaire interface for answering questions could be accessed as long as the participants clicked the option of “Agree”.

Finally, 658 questionnaires were collected, including 46 invalid or suspicious questionnaires and 612 valid questionnaires. The effective response rate reached 93.0%. This sample size met the common empirical rule for SEM, namely “having samples at least ten times the number of observed indicators for latent variables”. It also has sufficient statistical effects for subsequent confirmatory factor analysis (CFA) and path analysis.

Generally, the mentioned data collection process matched the research objectives in terms of scheduling, participant screening, ethical compliance, and quality control. The obtained data laid a reliable empirical foundation for exploring the emotional and psychological effects of AI virtual companion apps on long-term users. The demographic characteristics are presented in Table 4.

**Table 4:** Demographic characteristics.

**Item**	**Option**	**Frequency**	**Percentage, %**	**Cumulative percentage, %**
Gender	Male	295	48.2	48.2
	Female	317	51.8	100.0
Age	18–20 years old	140	22.9	22.9
	21–40 years old	178	29.1	52.0
	41–65 years old	152	24.8	76.8
	More than 65 years old	142	23.2	100.0
Education level	Inferior to junior college	147	24.0	24.0
	Junior college	146	23.9	47.9
	Master	165	27.0	74.8
	PhD	154	25.1	100.0
Occupation	Military/civil servant	141	23.0	23.0
	Agriculture/farming	170	27.8	50.8
	Industrial/blue-collar worker	148	24.2	75.0
	Business	153	25.0	100.0
Marital status	Single	116	19.0	19.0
	In love	126	20.6	39.5
	Married	118	19.3	58.8
	Divorced	131	21.4	80.2
	Other	121	19.7	100.0
Monthly income	Not more than RMB 3,000 Yuan	114	18.6	18.6
	RMB 3,001–6,000 Yuan	116	19.0	37.6
	RMB 6,001–10,000 Yuan	126	20.6	58.2
	RMB 10,001–30,000 Yuan	131	21.4	79.6
	More than RMB 30,000 Yuan	125	20.4	100.0
Commonly used AI virtual companion app	Talkie	123	20.1	20.1
	BagelBell	131	21.4	41.5
	Zhumengdao	122	19.9	61.4
	Soul	120	19.6	81.0
	Other	116	19.0	100.0
Duration of use to date	Within 1 month	115	18.8	18.8
	1–3 months	121	19.8	38.6
	3 months to 1 year	116	18.9	57.5
	1–3 years	145	23.7	81.2
	More than 3 years	115	18.8	100.0
Recent daily average time of usage	30 min	170	27.8	27.8
	31–60 min	129	21.1	48.9
	61–300 min	149	24.3	73.2
	More than 300 min	164	26.8	100.0

## 4 Data analysis

### 4.1 User interview analysis

After semi-structured interviews, the interview data from 10 long-term users were analyzed qualitatively to enrich the proposed “attachment, emotion, and behavior” model. This analysis strictly adhered to the six-step thematic analysis approach introduced by Braun and Clarke (2006): (1) getting familiar with the data, (2) generating initial codes, (3) searching for potential themes, (4) reviewing the themes, (5) defining and naming the themes, and (6) writing a report. The interview transcript texts were coded in an open format using NVivo 14, and 206 nodes were identified initially. After axial coding and aggregation, six core themes were refined, corresponding one-to-one with the six dimensions in the questionnaire (UF, EA, LON, SWB, SCC, and RSP). The user interview analysis result is presented in Table 5.

**Table 5:** User interview analysis.

**Dimension (code)**	**High-frequency themes in the interviews/ qualitative codes**	**Representative quotes**	**Questionnaire items supported/modified**	**Suggestions for the scale design**
AI usage frequency (UF)	- Daily routine: Morning/bedtime check-in - High interaction volume: >50 messages/day, >15 min/day - Sense of missing: Feeling like “something is missing” if not chatting for a day	“At first, I just wanted to play around, but later I started saying good morning and good night every day, just like a check-in routine.”, said P03	UF1, UF2, UF3, UF4, UF5	Using the mean value from interviews to calibrate the thresholds for “high/low” usage frequency
AI emotional attachment (EA)	- Sense of being understood: Customized personality → “It gets me” - Secure self-disclosure: Sharing privacy without misgiving - Separation anxiety: Feeling panic when offline and feeling sad when the service is stopped - Social visibility: Willingness to introduce it to friends	“I set it to the dialogue style in the movie *2046*, seeming it understands me better than my friends.”, said P11 “I would feel as sad as if I broke up with someone if the service is stopped.”, said P06	EA1-EA5	Retaining EA4 (feeling sad when service stopped) as a threshold for attachment intensity
Loneliness (LON)	- Before use: Lack of someone to talk to and social alienation - After use: Significant reduction in loneliness - Sense of being excluded: Reappearing when in conflict with reality	“Before using the AI companion, everyone in my friend circle was busy, and no one was available to chat; now, it's always ‘there' for me.”, said P09	LON1-LON5	Adding reverse-worded items
Subjective wellbeing (SWB)	- Enhancement of wellbeing: Living a nearly ideal life - Sense of purpose: Daily encouragement from the app → Increasing satisfaction	“It gives me ‘daily mini-goals,' and I feel a sense of accomplishment after completing them.”, said P04	SWB1-SWB5	Using a “pre-use vs. post-use difference” measurement approach
Self-identity clarity (SCC)	- Self-reflection: Conversations with AI can trigger introspection - Identity stabilization: Improving emotional control and self-efficacy - Occasional confusion: Making a blurred judgment when relying on AI's suggestions	“After rounds of talking to the AI companion, I become clearer about what I care about instead.”, said P07	SCC1-SCC5	Adding a reverse-worded item of “over-reliance → identity blur”
Real-life social participation (RSP)	- Positive pathway: Humor script training → Increasing offline initiative - Negative pathway: Conflict outsourcing → Enhancing face-to-face avoidance	“It taught me how to joke with colleagues, and now I'm more daring to speak offline.”, said P02 “In the face of argument, I retreat to the app because it never argues back.”, said P08	RSP1-RSP5	Adding a reverse-worded item of “social avoidance” in the scale to verify differences

### 4.2 Reliability analysis

Data selected from the scales were used for reliability and validity analyses. Cronbach's alpha was employed to assess the reliability of the selected data. In reliability analysis, a Cronbach's alpha coefficient exceeding 0.7 generally implies a high reliability of the questionnaire, justifying further in-depth analysis of the questionnaire. As displayed in Table 6, the Cronbach's alpha coefficients for both dimensions and the overall questionnaire exceeded 0.7; the Corrected Item-Total Correlation (CITC) values were greater than 0.4; the Cronbach's alpha coefficients calculated after item deletion were consistently lower than the original coefficients for their respective dimensions. Hence, the overall questionnaire had a high reliability, without items requiring deletion.

**Table 6:** Reliability analysis.

**Dimension**	**Item**	**Corrected item-total correlation**	**Cronbach's alpha if item deleted**	**Cronbach's alpha**	**Overall Cronbach's alpha**
Usage frequency	A01	0.733	0.867	0.891	0.856
	A02	0.730	0.868		
	A03	0.718	0.870		
	A04	0.750	0.863		
	A05	0.735	0.867		
Emotional attachment	B01	0.729	0.831	0.868	
	B02	0.681	0.843		
	B03	0.698	0.839		
	B04	0.672	0.845		
	B05	0.676	0.844		
Loneliness	C01	0.727	0.863	0.888	
	C02	0.720	0.865		
	C03	0.742	0.860		
	C04	0.718	0.865		
	C05	0.729	0.863		
Subjective wellbeing	D01	0.727	0.845	0.877	
	D02	0.715	0.848		
	D03	0.692	0.854		
	D04	0.701	0.852		
	D05	0.697	0.853		
Self-identity clarity	E01	0.700	0.839	0.868	
	E02	0.714	0.835		
	E03	0.698	0.839		
	E04	0.677	0.844		
	E05	0.671	0.846		
Real-life social participation	F01	0.679	0.836	0.863	
	F02	0.698	0.831		
	F03	0.677	0.836		
	F04	0.692	0.833		
	F05	0.669	0.838		

### 4.3 Validity analysis

Factor analysis was conducted to assess the validity of the questionnaire. In validity analysis, a KMO measure above 0.7 generally reflects the suitability for factor analysis of the questionnaire. As shown in Table 7, the KMO value derived from the test was 0.921 (greater than 0.7); Bartlett's test of sphericity yielded a significance level (Sig.) of 0.000 (smaller than 0.001), showing statistical significance at the 0.001 level. Hence, it was appropriate to conduct a factor analysis.

**Table 7:** KMO and Bartlett's test results.

**KMO measure of sampling adequacy**		**0.921**
Bartlett's test of sphericity	Approximate chi-square	9,638.282
	Degrees of Freedom (DOF)	435
	Significance level	0.000

As further analyzed and shown in Table 8, the total variance explained by the factors extracted from the Service Quality Scale was 67.233%. In other words, the factors had good explanatory power; the six extracted factors could fully retain the original data information. Meanwhile, the variance extracted by the first-factor loading without rotation was 30.169% (lower than 40%). This manifested that the questionnaire did not have serious common method biases.

**Table 8:** Total variance explained.

**Ingredient**	**Initial eigenvalues**			**Extract the squared load**			**Square sum of repeated loads**		
	**Amount to**	**Variance percentage**	**Accumulate%**	**Amount to**	**Variance percentage**	**Accumulate%**	**Amount to**	**Variance percentage**	**Accumulate%**
1	9.051	30.169	30.169	9.051	30.169	30.169	3.487	11.625	11.625
2	2.887	9.623	39.792	2.887	9.623	39.792	3.484	11.613	23.238
3	2.362	7.873	47.664	2.362	7.873	47.664	3.365	11.218	34.456
4	2.288	7.627	55.291	2.288	7.627	55.291	3.303	11.009	45.464
5	1.818	6.061	61.352	1.818	6.061	61.352	3.294	10.981	56.445
6	1.764	5.881	67.233	1.764	5.881	67.233	3.236	10.787	67.233
7	0.628	2.093	69.325						
8	0.565	1.882	71.207						
9	0.535	1.783	72.990						
10	0.521	1.735	74.725						
11	0.517	1.722	76.448						
12	0.505	1.684	78.132						
13	0.483	1.609	79.741						
14	0.475	1.582	81.323						
15	0.458	1.528	82.851						
16	0.439	1.465	84.316						
17	0.426	1.421	85.737						
18	0.415	1.384	87.121						
19	0.402	1.339	88.460						
20	0.378	1.261	89.721						
21	0.359	1.196	90.917						
22	0.350	1.166	92.083						
23	0.339	1.131	93.214						
24	0.326	1.088	94.302						
25	0.318	1.061	95.363						
26	0.300	0.999	96.362						
27	0.287	0.958	97.320						
28	0.280	0.933	98.253						
29	0.268	0.895	99.148						
30	0.256	0.852	100.000						

Extraction method: principal component analysis method.

As observed in Table 9, all items are within their corresponding pre-set dimensions. This explained that the questionnaire had good structural validity, and the data obtained from it could be used for further analysis. On the whole, the entire questionnaire had high reliability and validity, making it suitable for research analysis. The component matrix after rotation is presented in Table 9.

**Table 9:** Component matrix after rotation.

**Item**	**Component**					
	**1**	**2**	**3**	**4**	**5**	**6**
A01	0.783					
A02	0.777					
A03	0.800					
A04	0.818					
A05	0.799					
B01				0.808		
B02				0.757		
B03				0.767		
B04				0.750		
B05				0.738		
C01		0.810				
C02		0.798				
C03		0.834				
C04		0.789				
C05		0.796				
D01			0.799			
D02			0.786			
D03			0.747			
D04			0.768			
D05			0.763			
E01					0.770	
E02					0.768	
E03					0.764	
E04					0.770	
E05					0.724	
F01						0.754
F02						0.764
F03						0.740
F04						0.752
F05						0.741

Extraction method: principal component analysis method. Rotation method: Kaiser normalization with varimax rotation. The rotation converged after six iterations.

### 4.4 Descriptive statistical analysis of the variables

The variables were analyzed by descriptive statistical means. As observed in Table 10, the absolute kurtoses were less than 10, and the absolute skewness values were less than 3. Hence, the variables could be treated as in a normal distribution.

**Table 10:** Descriptive statistics of the variables.

**Dimension**	**Number of cases**	**Minimum**	**Maximum**	**Mean**	**Standard deviation**	**Skewness**	**Kurtosis**
Usage frequency	612	1.2	7	4.98	1.46	−0.890	−0.441
Emotional attachment	612	1.2	7	5.03	1.40	−0.867	−0.407
Loneliness	612	1.4	7	4.88	1.48	−0.771	−0.666
Subjective wellbeing	612	1.4	7	5.03	1.42	−0.849	−0.503
Self-identity clarity	612	1.4	7	5.10	1.35	−0.891	−0.367
Real-life social participation	612	1.2	7	5.11	1.33	−0.827	−0.530

As indicated in the table above regarding the questionnaire scale, the mean value was 4.98 for usage frequency, 5.03 for emotional attachment, 4.88 for loneliness, 5.03 for subjective wellbeing, 5.10 for self-identity clarity, and 5.11 for real-life social participation.

### 4.5 Correlation analysis

Pearson correlation analysis was conducted to explore the correlations among five variables (i.e., usage frequency, emotional attachment, loneliness, subjective wellbeing, self-concept clarity, and real-life social participation). The obtained Pearson correlation coefficients among the five mentioned variables and the significance levels are listed in Table 11.

**Table 11:** Correlation analysis.

**Dimension**	**Usage frequency**	**Emotional attachment**	**Loneliness**	**Subjective wellbeing**	**Self-identity**	**Real-life social participation**
Usage frequency	1					
Emotional attachment	0.347^**^	1				
Loneliness	−0.300^**^	−0.248^**^	1			
Subjective wellbeing	0.356^**^	0.311^**^	−0.317^**^	1		
Self-identity clarity	0.319^**^	0.411^**^	−0.209^**^	0.406^**^	1	
Real-life social participation	0.331^**^	0.428^**^	−0.296^**^	0.377^**^	0.441^**^	1

^*^The correlation is significant at the 0.01 level (two-tailed).

^*^p < 0.05; ^**^p < 0.01.

As observed, the significance levels of the correlations between all the variables were less than 0.01, indicating significant correlations. Specific analysis of the above table revealed the following points:

(1) The correlation coefficient between real-life social participation and usage frequency was 0.347, with a significance level of 0.01. This denoted a significant positive correlation between the two variables;
(2) The correlation coefficient between real-life social participation and emotional attachment was 0.428, with a significance level of 0.01. This suggested a significant positive correlation between the two variables;
(3) The correlation coefficient between real-life social participation and loneliness was −0.296, with a significance level of 0.01. This implied a significant negative correlation between the two variables;
(4) The correlation coefficient between real-life social participation and subjective wellbeing was 0.377, with a significance level of 0.01. This reflected a significant positive correlation between the two variables;
(5) The correlation coefficient between real-life social participation and self-concept clarity was 0.441, with a significance level of 0.01. This denoted a significant positive correlation between the two variables.

### 4.6 Confirmatory factor analysis

**Figure 3:**
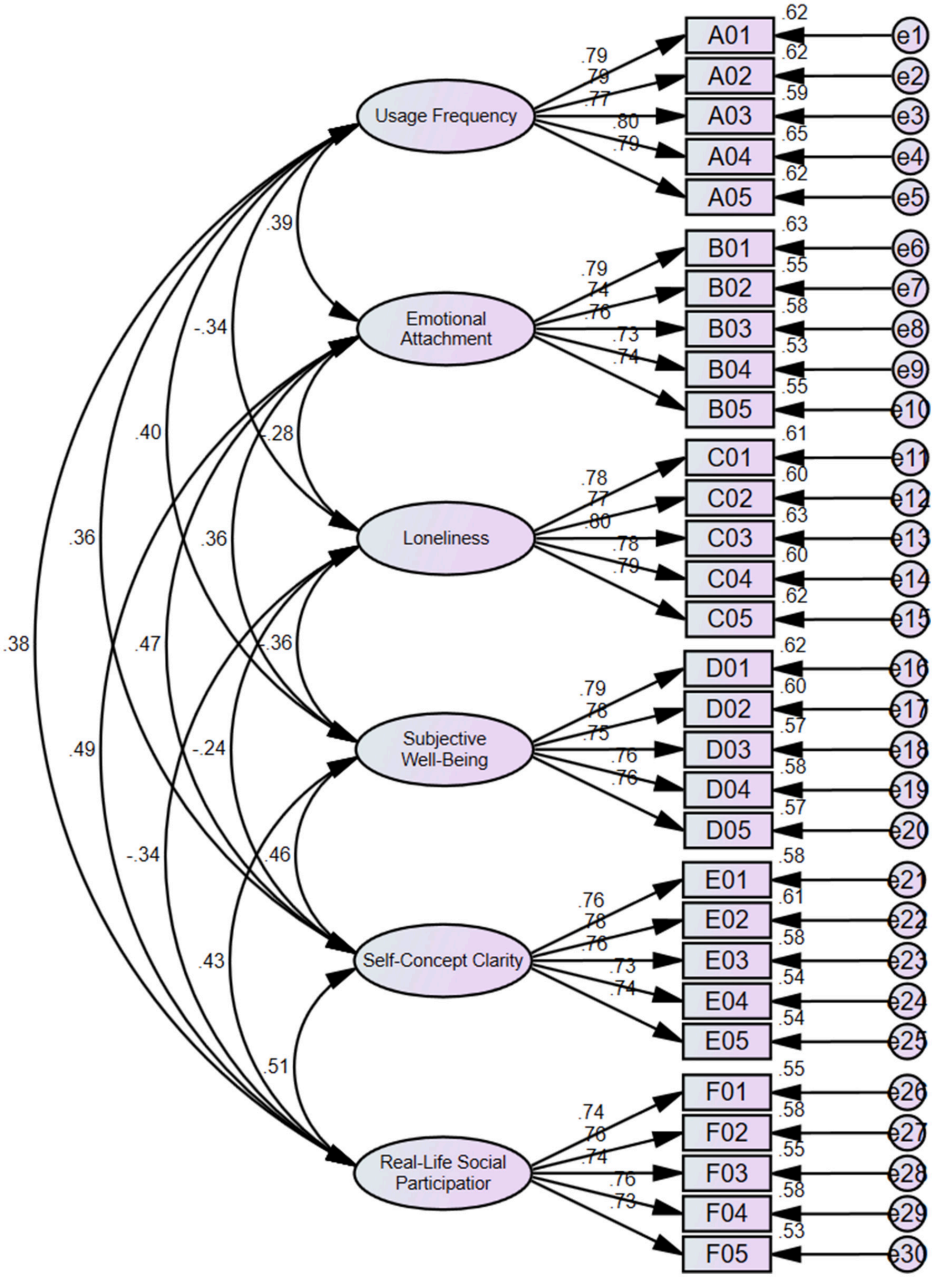
CFA diagram.

The questionnaire was further conducted with a CFA as illustrated in Figure 3. Generally, in CFA, the composite reliability (CR) and construct validity among data are reflected as high if the following conditions are met: normalized factor loadings >0.6, CR > 0.7, and average variance extracted (AVE) >0.5.

As observed from Table 12, the degrees of fitting from CFA met the ideal thresholds, proving the reliability of the analysis results.

**Table 12:** Confirmatory factor analysis.

**Model fit indices**	**CMIN/DF**	**GFI**	**AGFI**	**IFI**	**RMSEA**	**CFI**	**TLI**
Ideal value	≤ 3.00	≥0.90	≥0.90	≥0.90	≤ 0.08	≥0.90	≥0.90
Degree of fitting	1.406	0.945	0.934	0.983	0.026	0.983	0.981

CMIN/DF, Chi-square Minimum Discrepancy/Degrees of Freedom; GFI, Goodness-of-Fit Index; AGFI, Adjusted Goodness-of-Fit Index; IFI, Incremental Fit Index; RMSEA, Root Mean Square Error of Approximation; CFI, Comparative Fit Index; TLI, Tucker–Lewis Index.

As seen from Table 13, the normalized factor loadings of all items and the CR and AVE values of all dimensions met the criteria, exhibiting high CR and construct validity of the data.

**Table 13:** Normalized factor loadings.

**Dimension**	**Item no**.	**Normalized factor loading**	**CR**	**AVE**
Usage frequency	A01	0.790	0.891	0.620
	A02	0.788		
	A03	0.766		
	A04	0.803		
	A05	0.789		
Emotional attachment	B01	0.792	0.868	0.569
	B02	0.744		
	B03	0.762		
	B04	0.731		
	B05	0.742		
Loneliness	C01	0.780	0.888	0.613
	C02	0.775		
	C03	0.795		
	C04	0.776		
	C05	0.788		
Subjective wellbeing	D01	0.787	0.877	0.588
	D02	0.777		
	D03	0.752		
	D04	0.759		
	D05	0.757		
Self-identity clarity	E01	0.761	0.869	0.570
	E02	0.782		
	E03	0.761		
	E04	0.732		
	E05	0.737		
Real-life social participation	F01	0.739	0.863	0.558
	F02	0.763		
	F03	0.743		
	F04	0.760		
	F05	0.731		

### 4.7 Discriminant validity

At last, the square roots of AVEs for all dimensions were compared with the correlation coefficients between the dimensions. The results unveiled that the square roots of AVEs for all dimensions were greater than the correlation coefficients between the dimensions. This confirmed that the correlation within each dimension was stronger than that between dimensions, demonstrating good discriminant validity of the data. In summary, the data exhibited satisfactory reliability and validity and thus were suitable for further analysis. The discriminant validity of the data is presented in Table 14.

**Table 14:** Discriminant validity.

**Dimension**	**Usage frequency**	**Emotional attachment**	**Loneliness**	**Subjective wellbeing**	**Self-identity**	**Real-life social participation**
Usage frequency	**0.787**					
Emotional attachment	0.394	**0.754**				
Loneliness	−0.337	−0.277	**0.783**			
Subjective wellbeing	0.402	0.355	−0.360	**0.767**		
Self-identity clarity	0.362	0.473	−0.238	0.462	**0.755**	
Real-life social participation	0.378	0.490	−0.337	0.430	0.509	**0.747**

The bold values on the diagonal represent the square roots of the average variance extracted (AVE) for each construct. Discriminant validity is supported when the square root of AVE for a construct is greater than its correlations with other constructs.

### 4.8 Structural equation model (SEM)

The proposed hypotheses were validated through SEM. Specifically, the relationships between latent variables were analyzed based on the covariance matrix of the latent variables. SEM can simultaneously handle multiple dependent variables (endogenous variables). The regression coefficients in traditional regression models and the path coefficients in path analysis are calculated one by one for each dependent variable, regardless of the influence of other dependent variables. Yet, in structural equations, the presence of other factors is fully taken into account. Namely, the structure within each factor is adjusted, considering other co-existing variables. As such, change is reflected in both the relationship between factors and the internal structure of each factor.

**Figure 4:**
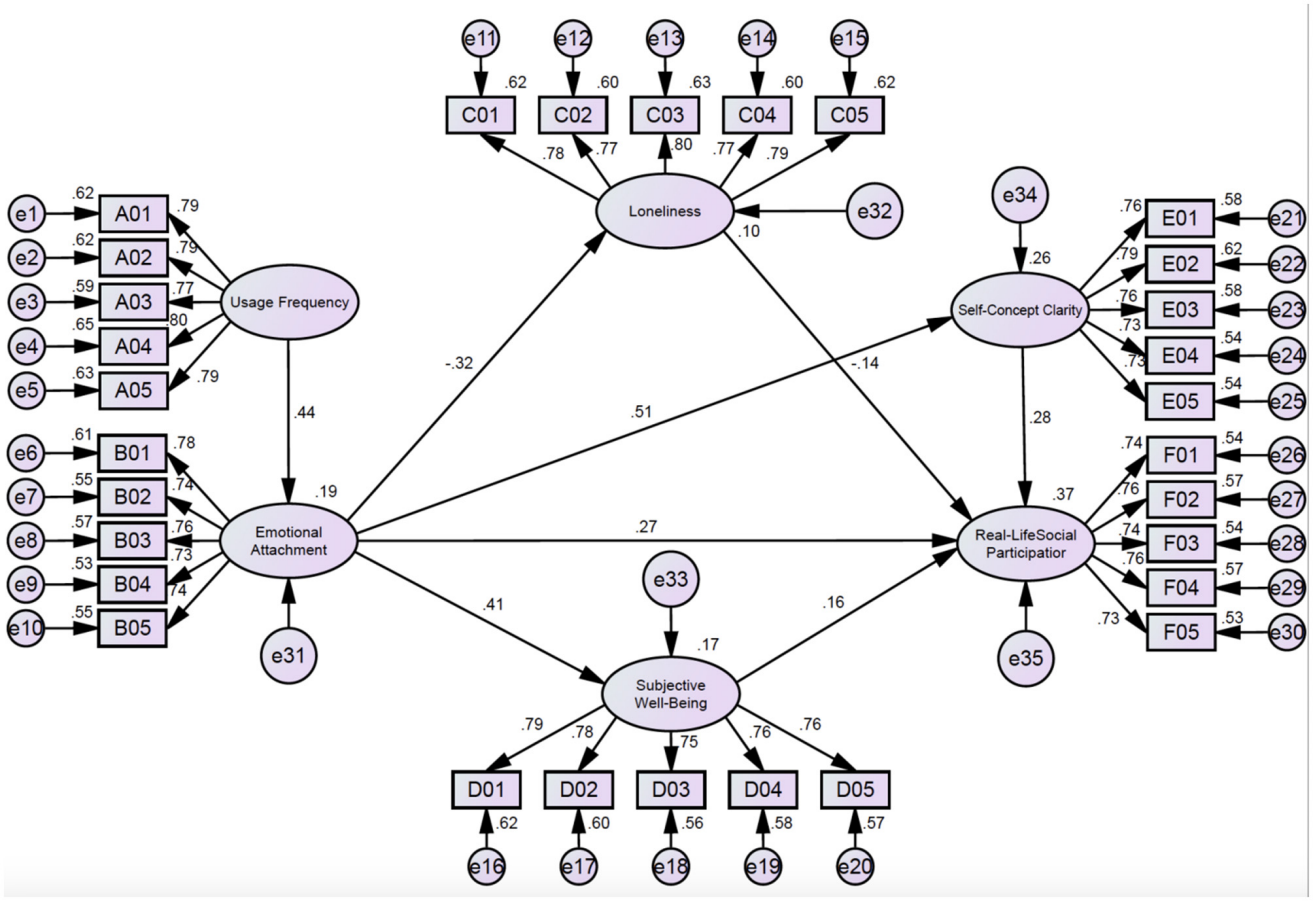
Diagram of path analysis.

First, the following hypotheses were established based on theories (Figure 4):

- H01: Usage frequency significantly and positively affects emotional attachment;
- H02: Emotional attachment significantly and negatively affects loneliness;
- H03: Emotional attachment significantly and positively affects subjective wellbeing;
- H04: Emotional attachment significantly and positively affects self-concept clarity;
- H05: Loneliness significantly and negatively affects real-life social participation;
- H06: Subjective wellbeing significantly and positively affects real-life social participation;
- H07: self-concept clarity significantly and positively affects real-life social participation;
- H08: Emotional attachment has an indirect, significant, positive impact on real-life social participation through loneliness;
- H09: Emotional attachment has an indirect, significant, positive impact on real-life social participation through subjective wellbeing;
- H10: Emotional attachment has an indirect, significant, positive impact on real-life social participation through self-concept clarity.

Structural equations were used to verify the path coefficients of the model. Consequently, all degrees of fitting for the model reached the ideal values, proving a good fit of the model. The path coefficients are listed in Table 15.

**Table 15:** Model fit indices.

**Fit indices**	**CMIN/DF**	**GFI**	**AGFI**	**IFI**	**RMSEA**	**CFI**	**TLI**
Ideal value	≤ 3.00	≥0.90	≥0.90	≥0.90	≤ 0.08	≥0.90	≥0.90
Degree of fitting	1.780	0.927	0.914	0.967	0.036	0.967	0.964

The path significance of SEM was analyzed using analysis of moment structures (AMOS), with results displayed in Table 16.

**Table 16:** Path analysis result.

**Path**	**se**	**Estimate**	**S.E**.	**C.R**.	***P***	**Hypothesis**
Usage frequency → Emotional attachment	0.438	0.396	0.043	9.276	^***^	Supported
Emotional attachment → Loneliness	−0.316	−0.341	0.050	−6.776	^***^	Supported
Emotional attachment → Subjective wellbeing	0.407	0.419	0.049	8.506	^***^	Supported
Emotional attachment → Self-identity clarity	0.507	0.497	0.048	10.346	^***^	Supported
Loneliness → Real-life social participation	−0.140	−0.120	0.036	−3.328	^***^	Supported
Subjective wellbeing → Real-life social participation	0.162	0.146	0.040	3.636	^***^	Supported
Self-identity clarity → Real-life social participation	0.277	0.263	0.047	5.589	^***^	Supported

^***^ indicates that the P-value is lower than 0.001.

As shown in Table 16, when the *P*-value was lower than 0.05, there was a significant difference in the path at the significance level of 0.05. The path influence coefficient of usage frequency on emotional attachment was 0.438, and the *P*-value of the significance level was lower than 0.05. This suggested that usage frequency positively affected emotional attachment. Therefore, H1 was supported.

The path influence coefficient of emotional attachment on loneliness was −0.316, and the *P*-value of the significance level was lower than 0.05. This indicated that emotional attachment negatively affected loneliness. Hence, H2 was supported.

The path influence coefficient of emotional attachment on subjective wellbeing was 0.407, and the *P*-value of the significance level was lower than 0.05. This reflected that emotional attachment positively affected subjective wellbeing. Hence, H3 was supported.

The path influence coefficient of emotional attachment on self-concept clarity was 0.507, and the P-value of the significance level was lower than 0.05. This proved that emotional attachment positively affected self-identity. Therefore, H4 was supported.

The path influence coefficient of loneliness on real-life social participation was −0.140, and the *P*-value of the significance level was lower than 0.05. This reflected that loneliness negatively affected real-life social participation. Hence, H5 was supported.

The path influence coefficient of subjective wellbeing on real-life social participation is 0.162, and the *P*-value of the significance level was lower than 0.05. This implied that subjective wellbeing positively affected real-life social participation. Therefore, H6 was supported.

The path influence coefficient of self-concept clarity on real-life social participation was 0.162, and the *P*-value of the significance level was lower than 0.05. This denoted that self-concept clarity positively affected real-life social participation. Hence, H7 was supported.

### 4.9 Mediating effect test

To accurately verify the mediating effect, 5,000 samples were conducted using the PROCESS Bootstrap method, with a confidence interval level of 95%. As observed in Table 17, the path “Emotional attachment → Loneliness → Real-life social participation” showed an indirect effect of 0.044 and a confidence interval [0.017, 0.073] excluding 0. This indicated the presence of an indirect effect, supporting the hypothesis that there was a mediating effect in this influence path. The path “Emotional attachment → Subjective wellbeing → Real-life social participation” exhibited an indirect effect of 0.066 and a confidence interval [0.022, 0.113] excluding 0. This verified the presence of an indirect effect, supporting the hypothesis that there was a mediating effect in this influence path. The path “Emotional attachment → self-concept clarity → Real-life social participation” showed an indirect effect of 0.141 and a confidence interval [0.084, 0.198] excluding 0. This proved the presence of an indirect effect, supporting the hypothesis that there was a mediating effect in this influence path. The results of the mediating effect test are provided in Table 17.

**Table 17:** Mediating effect test results.

**Path**	**Effect**	**95% CI**		***P*-value**	**Conclusion**
		**Lower limit**	**Upper limit**		
Emotional attachment → Loneliness → Real-life social participation	0.044	0.017	0.073	0.002	H8 is supported
Emotional attachment → Subjective wellbeing → Real-life social participation	0.066	0.022	0.113	0.005	H9 is supported
Emotional attachment → Self-identity clarity → Real-life social participation	0.141	0.084	0.198	0.000	H10 is supported
Total indirect effect	0.251	0.184	0.320	0.000	
Direct effect	0.273	0.161	0.389	0.000	
Total effect	0.524	0.443	0.599	0.000	

## 5 Discussion

Grounded in attachment theory, this study examined long-term users of AI virtual companions in mainland China to construct a conceptual model encompassing ‘usage frequency—emotional attachment—psychological state—real-world social engagement'. Using a mixed-methods approach that combined qualitative interviews with a large-scale cross-sectional survey, the study investigated the relationships between these variables. Overall, the findings support the associative patterns depicted in the predefined pathways and provide a deeper understanding of the five key dimensions.

### 5.1 Formation of emotional attachment and theoretical implications

This study examined a sample of long-term users of AI virtual companions in mainland China and found a significant positive correlation between usage frequency and emotional attachment (β = 0.438). This finding is consistent with the fundamental “proximity-security” principle of attachment theory (Feeney, 1995) and highlights a distinctive pathway for forming attachments in digital environments. AI virtual companions provide users with an “always-present” attachment figure through stable, algorithmically optimized responses; contextual memory construction; and personalized character customization.

Unlike the reciprocal interactions of traditional interpersonal attachments, this interaction model more closely resembles the “familiarity interaction and emotional investment” pathway summarized in human–computer relationship studies (Gillath et al., 2021). Users' attachment experiences often accumulate gradually through seemingly mundane daily routines, such as exchanging greetings in the morning and evening or having brief daily conversations. Over time, these repetitive behaviors become emotionally charged and serve as a significant source of attachment.

Crucially, this emotional bond is not merely a replication of traditional relationships, but exhibits distinct digital characteristics. Users actively participate in “co-creating their attachment figure” by customizing the AI companion's personality, backstory, and conversational style. This creative involvement further strengthens the emotional connection (Koles and Nagy, 2021). As P11 stated: “I set it to speak like the characters in 2046. It feels like it understands me better than my friends.” This remark highlights the unique value of AI virtual companions, which unconditionally accept the user's perceived ‘authentic self' while simultaneously embodying an idealized image of the other—a duality that is rarely fully realized in real-world relationships.

These findings complement attachment theory itself. Within the classical framework, attachment figures are generally considered to be individuals with “real existence” and “interactive capacity”. However, the present study shows that users can develop genuine emotional attachments to technological systems that consistently provide coherent and responsive interactive experiences. Therefore, rather than viewing digital attachment as a ‘degenerate form' of attachment, it should be understood as an adaptive extension of human emotional capacity in an era of widespread AI involvement.

### 5.2 Multidimensional psychological outcomes and their dynamic equilibrium

This study reveals a triple association between emotional attachment, loneliness, and subjective wellbeing, as well as between emotional attachment, loneliness, and self-concept clarity. This provides a more detailed framework for understanding the psychological impact of AI virtual companions. The negative correlation between emotional attachment and loneliness (β = −0.316) suggests that digital companionship may reduce perceptions of social isolation. This aligns with the World Health Organization's view that digital tools could play a positive role in addressing the “loneliness crisis” (Chaturvedi et al., 2024).

Concurrently, the positive correlations between emotional attachment and subjective wellbeing (β = 0.407) and self-concept clarity (β = 0.507) warrant particular attention. This suggests that digital attachment is associated not only with emotional comfort but also with deeper processes of self-understanding. Analysis of interviews revealed that AI virtual companions are often experienced as a “non-judgemental” space for self-exploration. Users can experiment with new emotional expressions, resolve internal conflicts and clarify personal values within this environment. Through repeated cycles of ‘testing—feedback—integration', users reported gradually developing a clearer and more coherent sense of self.

The structural model revealed that the association between self-concept clarity and real-world social participation (β = 0.277) was stronger than that with subjective wellbeing (β = 0.162). This highlights the pivotal role of identity-related processes in bridging the virtual and real worlds. When users gain a stronger sense of self-worth and value through interactions with AI virtual companions, this correlates with greater confidence and motivation to engage in real-world social participation. As P02 stated: ‘It taught me how to joke with colleagues, and now I speak up more in real life.'

However, these psychological outcomes are not unidirectionally positive. While alleviating loneliness can support mental health, overreliance on virtual companionship to fulfill social needs may diminish users' real-world social skills and willingness to engage (Nieves et al., 2024). As P08 noted: “When we argue, I retreat into this app because it never fights back.” This shows how virtual attachment can lead to a tendency to avoid conflict and risk offline withdrawal from challenging situations.

Therefore, the psychological impact of emotional attachment resembles a dynamic equilibrium. On the one hand, it can support emotional regulation, enhance wellbeing, and integrate self-concept. On the other hand, however, it may reinforce avoidance patterns, dependency or even identity vulnerability. When designing and using AI virtual companions, it is important to focus on promoting positive outcomes while mitigating these potential risks.

### 5.3 The “spillover” model and transformation mechanism of real-world social participation

Research findings suggest that emotional attachment has a positive ‘spillover' effect on real-world social participation via a series of psychological variables. This finding is consistent with the ‘digital extension' perspective of extended self-theory, supporting the idea that emotional attachment accumulates positive emotional resources (Zhang et al., 2024). In the SEM model of this study, emotional attachment was found to have significant indirect associations with real-world social participation via loneliness, subjective wellbeing, and self-concept clarity. The total indirect association accounts for approximately 47.9% of the model's total variance, reflecting the complex and nuanced relationship between virtual attachment and behaviors relating to real-world social participation.

Of the three mediating pathways, the indirect association via self-concept clarity (0.141) was strongest. This suggests a crucial psychological transformation: On the one hand, emotional attachment correlates with a clearer, more coherent self-concept, and on the other, this enhanced self-concept clarity relates to more positive real-world social engagement. From this perspective, AI virtual companions can be conceptualized as a “psychological gym”: users practice emotional expression, narrative integration, and social scripts in virtual environments, gaining experience and confidence in a relatively low-risk setting. These developed abilities and beliefs are then partially transferred to real-world contexts (Rabb et al., 2022).

Qualitative data provide ample evidence of this process. Many interviewees reported that they had learned to articulate their feelings more clearly, handle conflicts more calmly and initiate conversations more proactively through interactions with AI virtual companions. Some introverted users began participating in offline activities, while those with severe social anxiety reported feeling less fearful when speaking in group settings. Participants attributed these changes to the gradual accumulation of positive experiences and confidence gained within the virtual relationship.

However, this “spillover” effect does not occur naturally. Both existing research and our interviews suggest that positive transfer is more likely when users view AI virtual companions as practice partners or supplementary tools for real-world change. Conversely, when users perceive the companions as complete substitutes for real relationships and persistently avoid offline conflicts and challenges, their social skills may stagnate or even regress (Lu et al., 2025).

This suggests that AI virtual companion products should not simply fulfill emotional needs endlessly within a closed virtual loop. Instead, they should be designed to incorporate reality-oriented ‘transformation mechanisms', such as recommending offline activities, encouraging users to engage in conversation with real people and guiding them to reflect on how to apply virtual insights into real life.

### 5.4 Cultural contexts and local characteristics of emotional attachment

This study is situated within the context of mainland China, and its conclusions should be understood in this framework. Compared to existing research, which was primarily conducted in Western, individualistic cultural settings, the Chinese users in this study's sample exhibited certain collectivist and relationship-oriented characteristics when interacting with AI virtual companions.

First, many respondents did not view their AI virtual companions as entirely “personal secrets”, but rather attempted to integrate them into their existing social networks. For example, some participants introduced their AI virtual companions to friends or invited friends to experience the application together. This form of “social sharing” is relatively uncommon in Western research. It reflects the inclusivity and fluidity of “relationships” in Chinese culture, suggesting that AI virtual companions may have a stronger social function in this context (Liu et al., 2024).

Second, Chinese users generally emphasize the ‘educational' and ‘growth-oriented' functions of AI virtual companions. Many respondents expressed a desire for AI companions to offer emotional support and assist in acquiring knowledge, improving character flaws, and enhancing social skills. This growth-oriented motivation aligns with the cultural emphasis on “self-cultivation” and “self-improvement”, offering guidance on localized design—specifically, integrating learning and self-development features alongside emotional support (Jiang et al., 2022).

Third, in terms of emotional terminology, users more frequently describe their relationship with AI virtual companions using terms such as “confidant” or “partner”, rather than direct labels such as “lover”. This may reflect the relatively conservative attitude toward virtual intimate relationships in Chinese culture (Wu, 2024). Such cultural norms influence how attachment is expressed and interpreted, yet they do not alter the fundamental mechanisms of attachment. This partially supports the cross-cultural applicability of attachment theory.

### 5.5 Reflections on strategy and ethics

Based on the findings of this study, several comprehensive implications can be drawn across three dimensions: AI virtual companion product design, mental health services and social governance. It is also crucial to examine potential ethical and social risks in a more balanced manner.

Product design. AI virtual companion products can improve attachment experiences by offering features such as personalized schedule reminders, mood tracking, and conversation history management. However, it is crucial to explicitly embed mechanisms that connect to real life within the design. For example, once users have achieved a certain level of emotional fulfillment, the system could proactively suggest offline social opportunities or share information about local interest groups. It could also encourage users to apply the expression methods they have practiced online to offline interactions. Incorporating self-expression and co-creation modules, such as narrative sharing, value clarification and ‘emotional milestones', supports the development of a clear, positive self-concept, moving beyond mere short-term emotional comfort (McDaniel et al., 2025). At the same time, designers must guard against inadvertently fostering excessive dependency. For example, systems that consistently provide instant responses without challenging users' perspectives or rewarding prolonged isolated usage may foster emotional dependency and reduce opportunities for real-world interaction. Transparent feedback, moderate “gentle challenges”, and configurable “break reminders” can help users to maintain a healthier balance between digital engagement and real-life relationships.

Mental Health Services. AI virtual companions can serve as supplementary tools to traditional counseling and therapy under strict privacy protections. They are particularly useful for early intervention in loneliness, social anxiety, and depressive symptoms. Mental health professionals can analyze user interaction patterns with AI companions, such as the frequency of interaction, the emotional vocabulary used, and the topics avoided, to identify potential risks and provide personalized intervention recommendations. AI virtual companions can also provide mental health education by conveying knowledge and skills, such as emotional regulation and stress management, through daily conversations. However, there are clinical risks: If users become overly reliant on AI virtual companions and delay or substitute professional help, or if the AI system inadvertently reinforces harmful cognitive patterns and behaviors such as avoidance, rumination or idealization of virtual relationships, the overall psychological impact may be detrimental (Gao, 2024). Therefore, the development and use of AI virtual companions necessitates close collaboration with mental health professionals. This collaboration should establish clear safety boundaries, protect user data privacy, confirm ownership of digital assets, and transparently communicate the functional limitations of AI virtual companions to users.

Social governance. At the level of public policy, AI virtual companions could be incorporated into public mental health services to provide additional emotional support for children who have been left behind, elderly individuals who live alone, and people who experience social difficulties. However, corresponding regulations and ethical frameworks must also be established to address issues, such as data protection, emotional manipulation, algorithmic transparency, and the commercial exploitation of vulnerable users.

From a broader societal perspective, attention must be paid to whether the increasing use of AI virtual companions could subtly change norms around intimacy, caregiving, and social responsibility. For example, might people gradually lower their expectations of ‘mutual care' in real-world relationships, thereby shifting more emotional labor to technological systems? Public discourse and ethical guidance should encourage viewing AI virtual companions as supplementary resources rather than substitutes for human relationships.

## 6 Conclusion

Adopting an attachment theory perspective, this study systematically examines key psychological dimensions, including usage frequency, emotional attachment, loneliness, subjective wellbeing, and self-identity clarity, among users of long-term AI virtual companion applications. Using structural equation modeling, we comprehensively validate the direct and indirect effects of these factors on the willingness to engage in real-world social participation. The findings provide an empirical foundation for understanding human-AI emotional interactions and their social consequences, while offering crucial evidence for the potential role of virtual emotional technologies in providing psychological support and promoting social engagement.

A significant positive correlation was observed between high-frequency interaction and emotional attachment, with emotional attachment exhibiting sustained spillover effects across multiple psychological dimensions. Stronger emotional attachment alleviates loneliness while enhancing subjective wellbeing and self-identity clarity. These psychological effects collectively promote users' real-world social engagement, yielding dual outcomes: positive social momentum and the potential for emotional substitution. This suggests that AI virtual companions provide emotional compensation and profoundly shape users' self-perception and social motivation.

The research findings also provide practical guidance on how to optimize virtual companion products. For example, healthy emotional interactions can be strengthened through more consistent interaction rhythms and contextual memory mechanisms. For example, personalized emotion tracking, conversational continuity design and contextual feedback can all promote stable attachment experiences. Virtual interactions can also facilitate real-world social engagement. Once users have experienced stable emotional support, encouraging them to attend city events, join interest communities, or participate in offline experiences can extend these emotional connections into reality, thereby mitigating the risk of social withdrawal stemming from emotional compensation. Furthermore, supporting users' identity construction is crucial. Virtual companion systems can enhance users' sense of self-consistency by providing spaces for self-expression, activities for co-creating values, and feedback on symbolic achievements. When necessary, these systems should integrate with mental health resources to address potential risks arising from excessive dependency.

This study focuses on Chinese adult users who are long-term participants in digital interactive environments because they exhibit heightened sensitivity to technology in emotional regulation and identity formation. The findings provide evidence to support the use of AI emotional technologies to help high-stress or high-loneliness-risk groups, and also offer insights into the value of virtual emotional systems in psychosocial interventions, digital companionship services, and social facilitation.

Despite adopting a systematic methodological approach, this study has several limitations. First, the sample primarily originates from Chinese internet communities, where cultural orientations may influence the manifestation of attachment mechanisms. Second, cross-sectional surveys cannot reveal the dynamic evolution of attachment relationships. Future research could use longitudinal tracking or experimental methods to reveal the time-series characteristics of human–AI attachment. Cross-cultural studies could also help to validate the universality of the model. Integrating multi-source data, such as behavioral logs and physiological signals, will deepen our understanding of human–AI symbiotic relationships and provide more reliable evidence for the responsible development of AI affective technologies.

## Data Availability

The original contributions presented in the study are included in the article/supplementary material, further inquiries can be directed to the corresponding author.
